# Assessment of trabecular bone score, an index of bone microarchitecture, in HIV positive and HIV negative persons within the HIV UPBEAT cohort

**DOI:** 10.1371/journal.pone.0213440

**Published:** 2019-03-21

**Authors:** Tara McGinty, Aoife G. Cotter, Caroline A. Sabin, Alan Macken, Eoin Kavanagh, Juliet Compston, Gerard Sheehan, John Lambert, Patrick W. G. Mallon, on behalf of the HIV UPBEAT (Understanding the Pathology of Bone Diseases in HIV-infected Subjects) Study Group

**Affiliations:** 1 HIV Molecular Research Group, School of Medicine, University College Dublin, Dublin, Ireland; 2 Department of Infectious Diseases, Mater Misericordiae University Hospital, Dublin, Ireland; 3 Institute for Global Health, UCL, London, United Kingdom; 4 Cambridge Biomedical Campus, Cambridge, United Kingdom; Medical College of Wisconsin, UNITED STATES

## Abstract

**Introduction:**

Increased prevalence of low bone mineral density (BMD) and increased fracture incidence are observed in persons living with HIV (PLWH). The trabecular bone score (TBS) is a novel index of bone microarchitecture which improves fracture prediction independent of BMD.

**Methods:**

The HIV UPBEAT study is a single centre, prospective cohort study that enrolled subjects with and without HIV from similar sociodemographic backgrounds for annual assessments of bone health. TBS was derived from lumbar spine (LS) dual-energy X-ray absorptiometry images. Univariate and multivariable linear regression was used to assess relationships between baseline TBS, BMD, sociodemographic and clinical factors.

**Results:**

463 subjects (201 HIV positive) were included; PLWH were younger and more likely male, of non-African ethnicity and current smokers. HIV was associated with a mean reduction of 0.037 [-0.060, -0.013] (*p* = 0.002) in TBS. Lower TBS was also associated with male gender, non-African ethnicity, current smoking status and lower LS BMD. HIV remained associated with lower TBS after adjustment for LS BMD, age, gender and ethnicity. However, adjustment for current smoking significantly attenuated the association between HIV and TBS, with further adjustment for higher bone turnover markers largely explaining any residual association. Among the sub-group of PLWH, exposure to protease inhibitors and lower nadir CD4+ T-cell counts were both predictors of lower TBS.

**Conclusions:**

PLWH have lower TBS independent of LS BMD. However, this is largely explained by higher current smoking rates and higher bone turnover in those with HIV. Exposure to PI, but not tenofovir disproxil fumarate, also contributed to lower TBS in those with HIV.

## Introduction

Advances in HIV therapy and care have led to significant gains in life expectancy over the past decade [1]. This success has been accompanied by an increasing burden of non-AIDS and age-related co-morbidities such cardiovascular disease, kidney disease and bone disease [2–5].

Low bone mineral density (BMD) and fractures are prevalent in people living with HIV (PLWH). The aetiology of low BMD in HIV is multifactorial. Even after controlling for traditional risk factors such as smoking and low body mass index (BMI), which are over-represented in PLWH [6], HIV remains an independent risk factor for low BMD [6]. Fracture incidence is also approximately 50% higher in PLWH and fragility fractures have been reported to occur at a younger age in PLWH, on average, than in the general population [7–9]. It is also reported that the fracture risk algorithm FRAX may underestimate actual fracture risk in this population [10].

BMD is strongly related to bone strength, but does not capture all aspects of bone microarchitecture and composition. Until recently, bone microarchitecture could only be accurately evaluated by bone biopsy. Developments in imaging techniques now enable non-invasive assessment of bone structure—in PLWH, findings from high resolution peripheral quantitative CT (HR pQCT) studies have supported reports of a higher prevalence of low BMD in PLWH and also suggest poorer bone microarchitecture in this group [11]. However, HR pQCT is expensive and not readily accessible [12]. The trabecular bone score (TBS) is a novel imaging modality which is a relatively inexpensive and easily accessible indirect textural measure of bone microarchitecture. TBS uses variation in grey level texture between pixels from lumbar spine dual-energy X-ray absorptiometry (DXA) images to provide an indirect index of bone microarchitectural quality. Distorted microarchitecture results in greater grey level variation whereas, non-distorted areas of microarchitecture are represented by less variation. A larger variation in grey level between pixels results in lower TBS values and reflects weaker microarchitecture [13]. A TBS value of <1.2 (TBS is unit less) is considered indicative of degraded bone microarchitecture, a TBS of 1.2–1.34 of partially degraded microarchitecture and a TBS >1.35 of normal bone microarchitecture. TBS has been validated in the general population and has been shown to improve fracture prediction independent of BMD [14].

Despite the reported adjunctive utility of TBS as a measure of bone microarchitectural quality in the general population there are few reported data in PLWH [15–18]. Furthermore, the sociodemoographic and clinical factors which may predict altered TBS in PLWH have not been explored in a larger contemporary mixed cohort such as is represented in the ‘Understanding the pathology of bone disease in HIV Positive persons’ (HIV UPBEAT) cohort.

We aimed to examine whether HIV infection is associated with altered TBS and to determine relationships between sociodemographic and clinical factors with TBS in PLWH. In a sub-group analysis restricted to PLWH, we aimed to explore the HIV-specific factors which additionally contribute to altered TBS. We hypothesized that PLWH would have lower TBS even after adjustment for BMD, suggesting a weaker bone microarchitecture and increased skeletal fragility.

## Methods

### Patient population

The HIV UPBEAT cohort study is a single site, prospective, observational cohort that enrolled unselected sequential HIV-1 antibody positive subjects attending the Infectious Diseases outpatient department at the Mater Misercordiae University Hospital, Dublin, Ireland between 2011 and 2012 and unmatched HIV negative subjects from similar social, geographical and demographic backgrounds. The cohort was recruited in this manner to allow exploration of the relative contribution of all variables to the potential differences in bone health between those with and without HIV, while avoiding introduction of selection or matching bias. Subjects attended annual visits with data from the baseline visit contributing to this analysis. The study was approved by the Mater Miseercordiae University Hospital Institutional review board. All study subjects provided written informed consent.

### Clinical evaluation and laboratory analysis

Baseline study visits involved collection of demographic, socioeconomic, clinical and treatment information. Fasting blood tests including full blood count, renal profile, liver function tests and bone profiles, parathyroid hormone (PTH), serum 25-hydroxy vitamin D 25(OH)D and bone turnover markers serum (C-terminal cross-linking telopeptide of type I collagen (CTX), osteocalcin (OC) [N-mid osteocalcin] and procollagen type 1 propeptide (P1NP)) were recorded on all subjects. HIV-1 RNA and CD4+ T-cell counts were obtained for subjects with HIV. Methodologies and assays used in the analysis of laboratory samples, including acceptable coefficients of variation for each of the assays, have been previously described [6].

### Measurement of BMD and TBS

All participants underwent DXA scanning (Lunar Prodigy, GE Medical Systems, Wisconsin, USA) according to a standardised, study-specific protocol. BMD was recorded at total hip (TH), femoral neck (FN) and lumbar spine (LS) with LS BMD used for the present analysis. Low BMD was defined as a T-score between -2.5 and -1.0 in those over 40 years of age and a Z-score <-2 in those under 40 years old in accordance with the study protocol. TBS, derived from LS DXA images following extraction of the raw data using TBS insight software version 2.2.1 (Medimaps SA, France), was calculated as a single mean value using the same regions of interest used to measure BMD at lumbar vertebrae L1 to L4. All the subjects included in this analysis had a BMI within the validated range for TBS interpretation (BMI range within the cohort; 15–37 kg/m^2^).

### Statistical analysis

Descriptive statistics were expressed as median [interquartile range (IQR)] or number (percentage) as appropriate. Between-group differences were assessed using Wilcoxon or chi-square tests. Associations between variables of interest and TBS were explored using Spearman correlation. Independent associations between covariates and TBS were determined in the whole cohort using stepwise, multivariable linear regression, with models constructed to include variables in the following order: HIV status, LS BMD, age (per 5 year increment), gender, ethnicity (African/non-African), body mass index (BMI), smoking status (current/non-current smoker) and bone turnover markers. Given the collinearity of the bone turnover markers and alkaline phosphatase, these factors were each individually included in separate models. Note that whilst smoking status was initially categorised into three groups (current/ex-smoker/never smoker), only current smoking was found to be associated with lower TBS in initial descriptive analyses and therefore the two latter groups were combined for the purposes of the multivariate analysis. In sensitivity analyses, we also replaced current smoking status in the model with a measure of tobacco consumption (years of smoking), with similar findings. These results are therefore not reported.

We also conducted a further analysis restricted only to those subjects with HIV. Group comparisons were performed using Mann-Whitney tests or analysis of variance. Associations between TBS and HIV-specific covariates were explored using univariate followed by multivariable linear regression. Those variables with a p<0.1 on univariate testing were included in multivariable models. Models were constructed adjusting for LS BMD, age, gender, ethnicity, BMI and current smoking status followed by forward stepwise inclusion of HIV-specific factors. These were incorporated in order of best fit: acquisition risk, duration of known HIV infection, nadir CD4+ T-cell count and baseline antiretroviral therapy (ART) variables (duration of ART exposure, on tenofovir disproxil fumarate (TDF) at baseline, on a protease inhibitor (PI) at baseline, cumulative exposure to TDF, cumulative exposure to PI and number of ART regimens), with backward selection until a parsimonious model was achieved. The interaction between intravenous drug use (IVDU) and PI exposure at baseline was tested. A *p*-value of <0.05 was considered statistically significant. All analyses were performed using SAS version 9.3.

## Results

### Baseline characteristics of the cohort

Between February 2011 and July 2012, 474 subjects (210 (44%) HIV positive) completed a baseline study visit, of whom 463 (201 (43%) HIV positive) had baseline LS DXA available for TBS analysis. Baseline characteristics are detailed in Table 1. The HIV positive group were younger, more likely to be male, of African ethnicity and current smokers with a longer duration of tobacco consumption. BMI did not differ significantly between the groups. Hepatitis C infection was also more common in the HIV positive group (34 (16%)) compared to the HIV negative group (3 (1%)). Lumbar spine BMD (g/cm^2^) was significantly lower in the HIV positive versus the HIV negative group (1.176 [1.071, 1.304] vs. 1.237 [1.128, 1.341], *p*<0.0005). Markers of bone metabolism including corrected serum calcium, PTH and alkaline phosphatase (ALP) were all significantly higher in the HIV positive group (all *p*<0.005) as were biomarkers of bone formation (serum OC and P1NP) and resorption (serum CTx), (Table 1).

**Table 1:** Baseline characteristics of participants in the HIV UPBEAT cohort.

	HIV positive *n* = 201 Median [IQR] unless stated	HIV negative *n* = 262 Median [IQR] unless stated	*p*
Male *n* (%)	119 (59.2%)	114 (43.5%)	<0.001
Age (years)	39 [34, 46]	42 [35, 49]	0.057
African ethnicity *n* (%)	79 (39.3%)	65 (24.8%)	<0.001
BMI (kg/m^2^)	26 [23,30]	27 [24,30]	0.052
Current smoker *n* (%)	72 (35.8%)	44 (16.8%)	<0.001
Never smoked	93 (45.1%)	146 (55.7%)	<0.001
Ex-smoker	25 (19.1%)	55 (20.9%)	0.326
No. years smoking	20 [12–25]	15 [8–20]	0.045
Significant steroid exposure *n* (%)	4 (1.6%)	0	0.024
Hepatitis C infection	34 (16%)	3 (1%)	<0.001
Lumbar Spine BMD (g/cm^2^)	1.176 [1.071, 1.304]	1.237 [1.128, 1.341]	<0.001
TBS	1.349 [1.263, 1.436]	1.380 [1.301, 1.453]	0.009
Vitamin D (nmol/mL)	48 [32, 71]	51 [36, 71]	0.653
PTH (pmol/mL)	5.9 [4.5, 8.0]	5.3 [4.2, 6.9]	0.005
ALP (IU/mL)	78 [64, 103]	63 [53, 74]	<0.001
CA (mmol/mL)	2.30 [2.24, 2.36]	2.27 [2.22, 2.31]	<0.001
PO4 (mmol/mL)	1.05 [0.95, 1.16]	1.03[0.94, 1.14]	0.253
OC (ug/L)	21.95 [15.35, 28.37]	16.95 [13.39, 20.90]	<0.001
P1NP (ug/L)	49.3 [35.9, 65.8]	37.6 [28.9, 48.9]	<0.001
CTx (ug/L)	0.46 [0.30, 0.61]	0.34 [0.25, 0.45]	<0.001
**HIV Specific Factors:**		_	_
*Acquisition Risk*:		_	_
Heterosexual *n* (%)	107 (51%)	-	-
MSM *n* (%)	61 (29%)	-	-
IVDU *n* (%)	39 (18%)	-	-
Duration HIV disease (years)	4.5 [2, 8]	_	_
Nadir CD4 T-cell count (cells/mm^3^)	211[128,306]	_	_
Nadir CD4% T-cell count (%)	27 [21, 34]	_	_
Current CD4 T-cell count (cells/mm^3^)	471 [351, 664]	_	_
CD8 T-cell count (cells/mm^3^)	802 [589, 1108]	_	_
Current ART *n* (%)	182 (88%)	_	_
Cumulative years ART exposure (years)	2.7 [0.5, 5.0]	_	_
On TDF *n* (%)	152 (74%)	_	_
Cumulative exposure to TDF (years)	1.3 [0.1, 3.0]	_	_
On PI *n* (%)	91 (44%)	_	_
Cumulative exposure to PI (years)	0.3 [0, 2.4]	_	_

C.I—Confidence Interval, BMI—Body mass Index, BMD—Bone Mineral Density, Vitamin D— 25-OH- Vitamin D, PTH—Parathyroid hormone, ALP—Alkaline phosphatase, CA—Serum Calcium, PO4 —Serum phosphate, CTx—C-terminal cross-linking telopeptide of type I collagen, OC—osteocalcin, P1NP—procollagen type 1 propeptide, MSM—Men who have sex with men, IVDU—intravenous drug use, ART- antiretroviral therapy, TDF—Tenofovir disproxil fumarate, PI- Protease inhibitor

### TBS and demographics

**Fig 1:**
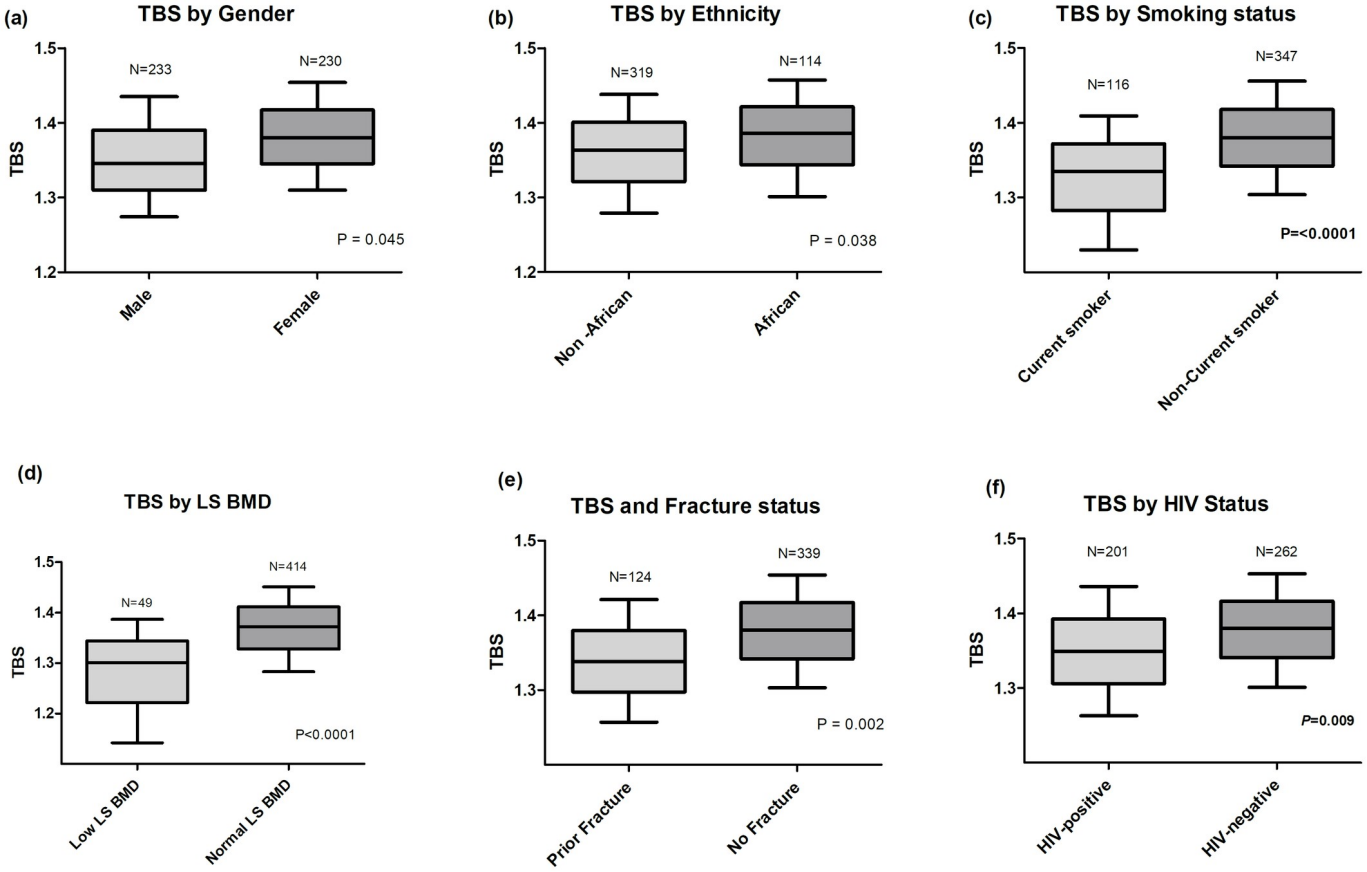
Median trabecular bone score according to (a) gender, (b). Ethnicity, (c). Smoking status, (d). Lumbar spine BMD, (e). Prior fracture history and (f). HIV status. TBS—Trabecular bone score, LS BMD—Lumbar spine bone mineral density, Low LS BMD defined as Z score < -2 in those 40 years old and under or a T score <-1.0 in those 40 years old or above.

The HIV positive group had significantly lower TBS compared to the HIV negative group (1.349 [1.263, 1.436] vs. 1.380 [1.301, 1.453], *p* = 0.009, Fig 1). In the cohort as a whole, significantly lower TBS values were associated with being male and of non-African ethnicity. Fig 1. Age was negatively correlated with TBS (*r* = -0.21, *p*<0.0001) as was having a diagnosis of Hepatitis C infection (*r* = -0.232, *p*<0.001).

### TBS and smoking

Being a current smoker was also associated with having a lower TBS compared to either ex-smokers (1.33 [1.20, 1.40] vs. 1.36 [1.29, 1.42], *p* = 0.03) or those who never smoked (1.33 [1.20, 1.4] vs. 1.38 [1.31, 1.46], *p* = 0.001) whereas there was no significant difference in TBS between ex-smokers and subjects who never smoked (*p* = 0.60). Tobacco consumption, measured by the number of reported years of smoking negatively correlated with TBS (*r* = -0.298, *p*<0.001).

### TBS, BMD and bone biomarkers

Within the cohort, 10.5% were identified with low LS BMD. In unadjusted analysis, TBS was lower in those with lower LS BMD (1.301 [1.142, 1.387] vs. 1.376 [1.298, 1.451], *p*<0.0001). Lower TBS correlated with lower BMD at all anatomical sites (LS BMD: *r* = 0.32, FN BMD: *r* = 0.32 and TH BMD: *r* = 0.33, all *p*<0.0001). Within laboratory variables, higher levels of ALP (*r* = -0.27, p<0.001), albumin (*r* = 0.17, *p* = 0.002), CTx (*r* = -0.45, *p* = 0.002), OC (*r* = -0.13, *p* = 0.002) and P1NP (*r* = -0.11, *p* = 0.02) correlated with lower TBS.

### Predictors of TBS within the cohort

In multivariable models, HIV status remained significantly associated with lower TBS after adjustment for LS BMD (Table 2, Model (i)) and further adjustment for age, gender, ethnicity and BMI (parameter estimate (PE) -0.029 [-0.058, 0.006], *p* = 0.01) (Table 2, model (ii)). However, further adjustment for current smoking status significantly attenuated the association between HIV status and TBS (PE -0.018 [-0.042, 0.006], *p* = 0.13, Table 2 model (iii)). However, current smoking status remained independently associated with lower TBS (PE -0.054 [-0.081, -0.027], *p*<0.0001, Table 2, model (iii)).

**Table 2:** Parameter estimates (effect of each parameter on the mean value) and 95% confidence intervals (95% C.I.) from a series of multivariable linear regression models to identify factors associated with TBS in the HIV UPBEAT cohort.

Model:	*(i)*			*(ii)*			*(iii)*			*(iv)*		
	Effect on TBS	95% C.I	*p*	Effect on TBS	95% C.I	*p*	Effect on TBS	95% C.I	*p*	Effect on TBS	95% C.I	*p*
HIV status (+ve vs.–ve)	-0.024	-0.046, -0.001	0.04	-0.029	-0.053, 0.006	0.01	-0.018	-0.042, 0.006	0.132	0.001	-0.024, 0.027	0.96
LS BMD (per 1g/cm^2^ increase)	0.003	0.184, 0.321	<0.001	0.002	0.002, 0.003	<0.001	0.002	0.002, 0.003	<.0001	0.200	0.130, 0.270	<0.001
Age (per 5 years increase)	-		-	-0.014	-0.020, 0.009	<0.001	-0.015	-0.021, -0.009	<.0001	-0.014	-0.020, -0.008	<0.001
Gender (male vs. female)	-	-	-	-0.0005	-0.024, 0.023	0.96	0.002	-0.022, 0.025	0.876	-0.0004	-0.025, 0.024	0.97
Ethnicity (African vs. non-African)	-	-	-	0.0004	-0.027, 0.028	0.97	-0.013	-0.041, 0.015	0.371	-0.014	-0.042, 0.014	0.32
BMI (per 10kg/m2 increase)	-	-	-	0.006	-0.017, 0.030	0.59	0.0006	-0.022, 0.024	0.958	0.007	-0.015, 0.031	0.50
Current Smoker vs. non-current smoker	-	-	-	-	-	-	-0.054	-0.081, -0.027	<.0001	-0.041	-0.067, -0.013	0.004
Albumin (per 5g/L increase)	-	-	-	-	-	-	-	-	-	0.019	0.002, 0.036	0.03
ALP (per 5IU/L increase)	-	-	-	-	-	-	-	-	-	-0.004	-0.006, -0.002	<0.001

Model (i) Adjusted for HIV status and Lumbar Spine BMD, (ii). Adjusted for HIV status, lumbar spine BMD, demographics and BMI, (iii). Further adjusted for smoking status, (iv). Additional adjustment for bone biomarkers

TBS—Trabecular bone score C.I—Confidence Interval, HIV status denotes HIV- positive versus HIV -negative status, LS BMD—Lumbar spine bone mineral density, BMI—body mass index, ALP—Alkaline phosphatase.

Finally, further adjustment for laboratory markers, specifically albumin and alkaline phosphatase (ALP), fully attenuated the association between HIV and TBS (PE 0.001 [-0.024, 0.027], *p* = 0.96, Table 2, model (iv)), without significantly influencing the association between current smoking status and lower TBS (PE -0.040 [-0.068, 0.013], *p* = 0.005). Substituting ALP with each of the bone turnover markers separately, CTx, OC and P1NP resulted in similar results to this model including ALP. (S1 Table).

### HIV sub-analysis

#### TBS in HIV positive persons

**Fig 2:**
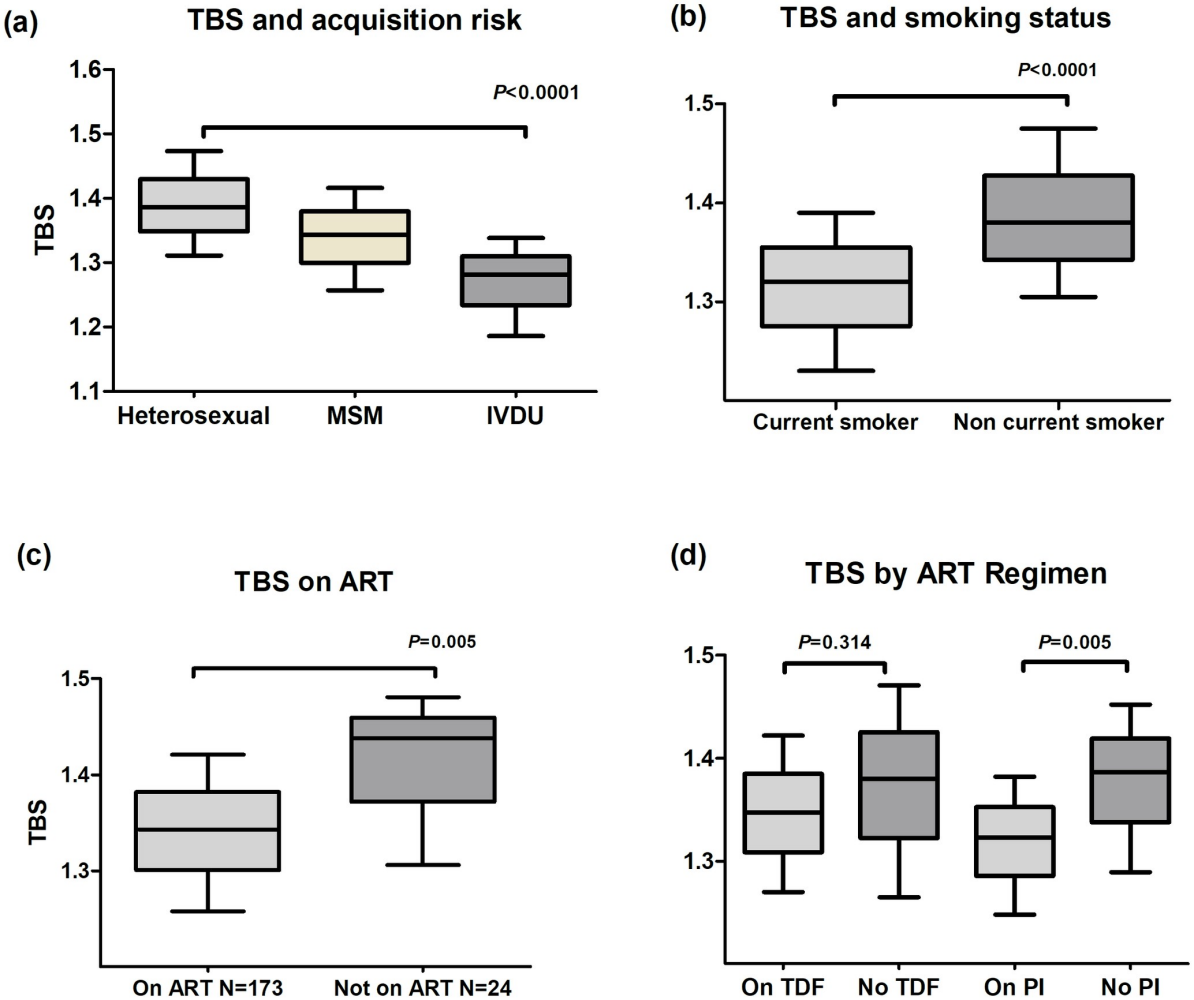
Median TBS according to (a). HIV acquisition risk, (b). Smoking status, (c). ART exposure and (d). TDF or PI exposure. TBS- Trabecular bone score, MSM—Men who have sex with men, IVDU—Intravenous drug user, ART—Antiretroviral therapy, TDF—Tenofovir disproxil fumarate, PI—Protease Inhibitor.

In analyses restricted to study subjects with HIV, TBS was lower in those who acquired HIV through IVDU compared to sex between men, or sex between men and women (1.282 [1.186, 1.338] vs. 1.343 [1.257, 1.416] and 1.386 [1.311, 1.473], respectively, *p*<0.0001). Notably, both Hepatitis C co-infection (n = 34 (87.1%)) and current smoking status (n = 31 (79.4%)) were over-represented within this HIV positive IVDU group. Lower TBS was also observed in current smokers compared to non-current smokers (1.320 [1.230, 1.390] vs. 1.380 [1.305, 1.475], *p*<0.0001) and those on ART compared to those not on ART (1.343 [1.258, 1.421] vs. 1.438 [1.306, 1.481], *p* = 0.005), Fig 2.

#### TBS and HIV-specific factors

In univariate analyses within the group with HIV, lower TBS was associated with IVDU (PE -0.117 [-0.163, -0.070], *p*<0.001), a longer known duration of HIV infection (PE -0.005 [-0.008, -0.002], *p* = 0.001), being Hepatitis C positive (PE -0.221, [-0.347, -0.097], *p* = 0.001), a lower nadir CD4+ T-cell count (PE per 50 cells/mm^3^ lower -0.005 [-0.003, -0.002], *p* = 0.004), exposure to a longer duration of ART (PE per year -0.018 [-0.036, 0.003], *p* = 0.01) and any exposure to PIs (PE -0.051 [-0.087, -0.015], *p* = 0.006) but not exposure to TDF (PE -0.023 [-0.065, 0.019], *p* = 0.27) (S2 Table).

#### HIV-specific predictors of TBS

In multivariable models adjusted for age, gender, ethnicity and BMI, both lower LS BMD (PE 0.214 [0.080, 0.394], *p* = 0.003) and current smoking status (PE -0.047 [-0.085, -0.008], *p* = 0.014) remained independently associated with lower TBS.

Following the above adjustments to all subsequent models, we included IVDU as an acquisition risk followed by known duration of HIV disease; in these analyses only IVDU was associated with lower TBS (PE -0.053 [-0.105, -0.002], *p* = 0.04). Next we included nadir CD4+ T-cell count which was an independent predictor of lower TBS (PE 0.006 [0.0002, 0.011], *p* = 0.04). Subsequently, on adjustment for exposure to PIs, the initial association between IVDU and lower TBS was fully attenuated (PE -0.036 [0.087, 0.014], *p* = 0.158). Furthermore, there was no significant interaction observed between IVDU and being on a PI at baseline.

A final parsimonious model was reached which best explained lower TBS within the HIV+ subgroup (Table 3, *R*^*2*^ = 0.32), including exposure to a PI-containing regimen (PE -0.045 [-0.079, -0.011, -0.0008], *p* = 0.009) and nadir CD4+ T-cell count (per 50 cells/mm^3^ lower) (PE 0.005 [0.0003, 0.011], *p* = 0.04)). Further models including adjustment for the number of ART regimens, cumulative exposure to ART or cumulative PI exposure did not significantly alter these findings, S2 Table.

**Table 3:** Final multivariable model: Predictors of TBS in HIV positive subjects in the HIV UPBEAT cohort.

Co-variates	Effect on TBS	95% C.I	*P*
LS BMD ((per 1g/cm^2^ increase)	0.002	0.001, 0.003	<0.001
Age (per 5 years increase)	-0.018	-0.027, -0.009	0.002
Gender (Male v Female)	-0.004	-0.044, 0.034	0.81
Ethnicity (African versus non-African	0.035	-0.009, 0.070	0.16
Current smoker	-0.047	-0.085, -0.008	0.017
Nadir CD4 T–cell count (per 50cells/mm)	0.005	0.0003, 0.011	0.040
On PI at baseline	-0.045	-0.079, -0.011	0.009

Table 3 **Legend**: HIV-related variables were added stepwise with predictors of TBS included in the subsequent steps in building the final model; this represents the final model which best predicts lower TBS in HIV.

LS BMD—Lumbar spine BMD, TBS—Trabecular bone score, C.I—Confidence Interval, IVDU—Intravenous drug use, PI—Protease inhibitor

## Discussion

This is one of the largest studies examining TBS in a diverse cohort of subjects with and without HIV recruited from similar sociodemographic and geographical backgrounds. We demonstrate that lower TBS in those with HIV is largely driven by current smoking status and altered bone turnover. Additionally, within the HIV group, exposure to PIs, but not TDF, and lower nadir CD4+ T-cell count were both independently associated with lower TBS. This finding highlights the dual contribution of immune status and exposure to ART to bone health.

The differences in TBS between those with and without HIV are consistent with results in prior studies using different modalities of microarchitectual bone measurement such as HR pQCT. In premenopausal HIV positive women and men on ART, abnormal bone microarchitecture, measured by HR pqCT, has been reported [19, 20]. Two other studies have assessed TBS in both those with and without HIV with differing results. In the Women’s Interagency HIV Study (WIHS), TBS was lower in HIV positive women (n = 319) compared to HIV negative women (n = 118), the HIV positive women being 64% more likely to have an abnormal TBS (less than 1.35) [18]. In this study, the association between HIV and TBS remained significant irrespective of HCV positive status. However, another recent study examining four groups of men (HIV mono-infected, HCV mono-infected, HIV/HCV co-infected and HIV negative controls), reported that HCV but not HIV predicted lower TBS [15]. While we also observed lower TBS in subjects with HCV, further comparisons were not explored within this analysis given the low prevalence of HCV in our cohort. However, taken together with the TBS data from HIV UPBEAT, these previous data suggest that assessment of TBS may be an important adjunct in the assessment of bone health in PLWH. Further larger studies are however, required to confirm this and to further explore the contribution of co-infection with HCV to lower TBS.

Another striking finding of our study was the association between current smoking status and TBS, which persisted after adjustment for demographics, BMD and bone turnover markers. We observed that the association was driven by current tobacco use rather than previous or cumulative tobacco exposure in our cohort. Smoking has been linked to lower BMD and increased fracture risk in the general population [21], but data linking smoking to bone microarchitecture are less robust. In the WIHS, current smoking was associated with a 57% increased likelihood of having an abnormal TBS although, contrary to our findings, HIV remained independently associated with lower TBS despite adjustment for smoking status. This is a uniquely female cohort with 75% of the cohort being of African or Hispanic ethnicity and over a quarter of the HIV positive women being postmenopausal, which may partly explain the differing results. A further large retrospective cohort of >29,000 postmenopausal women examined clinical predictors of TBS [22], using chronic obstructive pulmonary disease (COPD) as a surrogate marker of ever having smoked. Those with COPD had a 68% increased risk of having a TBS in the lowest versus highest tertile [21, 22]. Finally, a prospective cohort of over 800 older men reported microarchitectural deterioration as measured by HR pQCT in current smokers irrespective of demographic factors and BMD. These data underline the importance of smoking as a risk factor for bone health, which in our cohort explains much of the difference in TBS attributed to HIV in unadjusted analyses.

After correcting for current smoking status, the small residual association between HIV and lower TBS in our cohort was explained by higher bone turnover in those with HIV. Elevated bone turnover in PLWH has been reported in several studies and has been observed to be associated with lower BMD, lower nadir CD4+ T-cell counts, increased evidence of inflammation and ART-related factors [23]. Whilst there have been reports of associations between elevated bone turnover and lower TBS in other patient populations these relationships have yet to be fully explored in PLWH [24]. Although increased bone turnover coinciding with ART initiation has been observed in a number of studies [25–29], relationships between ART initiation, bone turnover and TBS have not been well described. In a single pilot study in 40 ART naïve subjects initiating ART (TDF/emtricitabine/elvitegravir/cobicistat),there was a significant decrease in TBS and BMD over 48 weeks, in association with increased bone turnover markers[16]. In light of these findings, in addition to our own, this merits further investigation.

In our study current use of PI but not TDF was independently associated with lower TBS in adjusted analyses, findings which are consistent to those reported in other studies. In a Spanish cohort examining TBS in over 300 PLWH, those on PI-containing ART had lower TBS than those on non-PI containing ART [30]. A further study of 64 PLWH on stable ART showed no significant differences in TBS between those on TDF or the alternative nucleoside reverse transcriptase inhibitor (abacavir) after 5 years on treatment.[17] Together, these data support the hypothesis that whilst TDF is associated with greater changes in BMD at ART initiation, there are no robust data to suggest a lower TBS in those on TDF, although longitudinal studies are required to confirm these findings.

We observed that PLWH who acquired HIV through IVDU had lower TBS than other acquisition risk groups. This acquisition risk has been observed to be associated with low BMD [31] but relationships between IVDU risk acquisition and TBS have not previously been reported. The association between IVDU risk acquisition and lower TBS was no longer significant when analyses were adjusted for PI use. This likely reflects a population in which multiple factors such as smoking, more frequent use of PIs and hepatitis C co-infection, all of which may contribute to both low BMD and low TBS, are over-represented. In addition other factors, not recorded in our analysis, for example lifestyle and dietary factors, may also contribute to poorer bone health in this vulnerable group.

Several studies have previously reported relationships between nadir CD4+ T-cell count and lower BMD [27, 32–34]. We observed similar relationships between lower nadir CD4+ T-cell counts and lower TBS. While several mechanisms have been proposed to explain relationships between immunodeficiency in PLWH and bone health, including increased immune activation and increased levels of circulating inflammatory cytokines which may drive increased bone turnover [27, 33, 35–38], data on TBS and baseline immune status are lacking. However, it is possible that the mechanisms involving increased immune activation and inflammation may also contribute to the observed alterations in TBS.

Our analysis has several limitations. The cross-sectional design prevents determination of causality. In addition, although UPBEAT does record a comprehensive set of socio-demographic, disease and treatment-specific information, the analysis may still be prone to unmeasured bias. A lack of prospective fracture data also limits the exploration of relationships between fracture and TBS and is beyond the scope of the current analysis. Furthermore we were not able to include data on use of vitamin D or calcium supplementation or detailed information on body fat distribution/ composition, all of which may influence bone turnover and / or TBS. There were no subjects on any specific anti-osteoporotic medications such as bisphosphonates.

Despite these limitations, the HIV UPBEAT cohort has the advantage of including a diverse cohort, with representation from African and Caucasian ethnicities, both genders and from all major HIV acquisition risk groups. The HIV positive groups have been treated with a number of different antiretroviral regimens. There is also a relevant HIV negative control group within our cohort drawn from similar geographical and sociodemographic backgrounds, which is often lacking in other HIV cohorts. In addition, through prospective collection of a robust dataset, including disease, lifestyle and sociodemographic factors, our results provide the basis for some novel insights into bone health in PLWH.

In conclusion, lower TBS, an index of bone microarchitectural quality, observed in our cohort of PLWH, is largely explained by current smoking and alterations in bone turnover, with PI exposure, but not TDF, and baseline immune status both contributing. That current smoking was a strong predictor of altered bone quality reinforces the benefit of smoking cessation as an important lifestyle intervention to reduce co-morbidity, including bone disease, in PLWH. Further studies on the clinical utility and role of TBS in fracture prediction in PLWH are merited.

## Supporting information

S1 Table Multivariable model; Predictors of TBS in HIV UPBEAT cohort including bone turnover markers. Model (i) CTx, (ii) OC and (iii) P1NP (all models are adjusted for the individual bone turnover marker in addition to adjustment for age, gender, ethnicity and BMI). HIV status denotes HIV- positive versus HIV -negative status, TBS—Trabecular bone score C.I—Confidence Interval, CTx—C-terminal cross-linking telopeptide of type I collagen, OC–osteocalcin, P1NP—procollagen type 1 propeptide.
(DOCX)

S2 Table Unadjusted and adjusted analysis within the HIV positive subgroup exploring associations between HIV-specific factors and lower TBS. *All variables adjusted for age, gender, Ethnicity, body mass index and current smoking status. Variables which remained significant following these adjustments remained in subsequent models until association was lost and the final model which best predicted lower TBS was reached, i.e. All variables after nadir CD4T-cell count had this variable in the model, IVDU remained in the models until the inclusion of PI exposure and was excluded from subsequent models as the association was lost, PI exposure was included in all models subsequent to its inclusion. ^ IVDU versus non IVDU adjusted for age, gender, Ethnicity, body mass index and current smoking status, nadir CD4 T-cell count and On Protease Inhibitor. TBS—Trabecular bone score, ART—Antiretroviral therapy, C.I—Confidence Interval, IVDU—Intravenous drug use, PI—Protease inhibitor, TDF—Tenofovir disproxil fumarate.
(DOCX)

S1 File Further supplementary tables and multivariable models associated with the data presented. Trabecular Bone Score (Median [IQR]); Between-group comparisons (Table A). Unadjusted HIV Effect on Trabecular Bone Score (Table B). Multivariable Models of Independent predictors of TBS (Models i to viii) (Table C). Multivariable model: Predictors of lower TBS using duration of smoking in years as a surrogate for tobacco consumption (Table D).
(DOCX)
