# Supplementary material for: Assessment of trabecular bone score, an index of bone microarchitecture, in HIV positive and HIV negative persons within the HIV UPBEAT cohort
Source: PLoS One. 2019 Mar 21;14(3):e0213440. doi: 10.1371/journal.pone.0213440 (PMC6428393; doi:10.1371/journal.pone.0213440)
Supplement: S1 Table — Model (i) CTx, (ii) OC and (iii) P1NP (all models are adjusted for the individual bone turnover marker in addition to adjustment for age, gender, ethnicity and BMI). HIV status denotes HIV- positive versus HIV -negative status, TBS—Trabecular bone score C.I—Confidence Interval, CTx—C-terminal cross-linking telopeptide of type I collagen, OC–osteocalcin, P1NP—procollagen type 1 propeptide. (DOCX) [file pone.0213440.s001.docx]

**Supplementary table 1: Multivariable model; Predictors of TBS in HIV UPBEAT cohort including bone turnover markers**

| Model | (i)  Effect on TBS | 95% C.I | *P* | (ii)  Effect on TBS | 95% C.I | *P* | (iii)  Effect on TBS | 95% C.I | *P* |
| --- | --- | --- | --- | --- | --- | --- | --- | --- | --- |
| HIV status | -0.005 | -0.031, 0.019 | 0.66 | -0.006 | -0.031, 0.020 | 0.66 | -0.006 | -0.031, 0.019 | 0.65 |
| Smoking status | 0.042 | -0.071, -0.015 | 0.002 | -0.040 | -0.068, -0.013 | 0.005 | -0.040 | -0.068, -0.012 | 0.005 |
| Albumin (per 5g/L increase) | 0.026 | 0.009, 0.043 | 0.002 | 0.027 | 0.010, 0.044 | 0.002 | 0.026 | 0.009, 0.043 | 0.002 |
| CTx (per 0.1 g/L increase) | -0.004 | 0.010, 0.001 | 0.133 | - | - | - | - | - | - |
| OC (per 5 g/L increase) | - | - | - | -0.004 | -0.010, 0.002 | 0.18 | - | - | - |
| P1NP (per 10 g/L increase) | - | - | - | - | - | - | -0.004 | -0.008, 0.0002 | 0.06 |

Model (i) CTx, (ii) OC and (iii) P1NP (all models are adjusted for the individual bone turnover marker in addition to adjustment for age, gender, ethnicity and BMI)

HIV status denotes HIV- positive versus HIV -negative status, TBS – Trabecular bone score C.I – Confidence Interval, CTx – C-terminal cross-linking telopeptide of type I collagen, OC –osteocalcin, P1NP – procollagen type 1 propeptide
