# Supplementary material for: Assessment of trabecular bone score, an index of bone microarchitecture, in HIV positive and HIV negative persons within the HIV UPBEAT cohort
Source: PLoS One. 2019 Mar 21;14(3):e0213440. doi: 10.1371/journal.pone.0213440 (PMC6428393; doi:10.1371/journal.pone.0213440)
Supplement: S2 Table — *All variables adjusted for age, gender, Ethnicity, body mass index and current smoking status. Variables which remained significant following these adjustments remained in subsequent models until association was lost and the final model which best predicted lower TBS was reached, i.e. All variables after nadir CD4T-cell count had this variable in the model, IVDU remained in the models until the inclusion of PI exposure and was excluded from subsequent models as the association was lost, PI exposure was included in all models subsequent to its inclusion. ^ IVDU versus non IVDU adjusted for age, gender, Ethnicity, body mass index and current smoking status, nadir CD4 T-cell count and On Protease Inhibitor. TBS—Trabecular bone score, ART—Antiretroviral therapy, C.I—Confidence Interval, IVDU—Intravenous drug use, PI—Protease inhibitor, TDF—Tenofovir disproxil fumarate. (DOCX) [file pone.0213440.s002.docx]

|  | *Unadjusted analysis* | | | *Adjusted Analysis** | | |
| --- | --- | --- | --- | --- | --- | --- |
|  | **Effect on TBS** | **95% C.I** | ***P*** | **Effect on TBS** | **95% C.I** | ***P*** |
| Duration HIV disease (years) | -0.005 | -0.008, -0.002 | 0.001 | -0.0005 | -0.004, 0.003 | 0.759 |
| IVDU versus non IVDU | -0.117 | -0.163, -0.070 | <0.0001 | -0.056 | -0.104, -0.007 | 0.024 |
| IVDU versus non IVDU^^^ |  |  |  | -0.037 | -0.088, 0.014 | 0.158 |
| Nadir CD4 T-cell count (cells/mm^3^) | 0.0001 | 0.0006, 0.0003 | 0.004 | 0.005 | 0.0001, 0.011 | 0.042 |
| CD8 T-cell count (cells/mm^3^) | 0.0001 | 0.0003, 0.0005 | 0.563 | - | - | - |
| Time with CD4 T-cell count<200(cells/mm^3^) | -0.022 | -0.040, -0.005 | 0.012 | -0.002 | -0.020, 0.015 | 0.809 |
| HIV RNA <40 | 0.008 | -0.031, 0.048 | 0.687 | - | - | - |
| On ART versus not on ART | -0.065 | -0.120, -0.009 | 0.022 | -0.023 | -0.079, 0.034 | 0.428 |
| On P.I versus not on PI | -0.051 | -0.087, -0.015 | 0.006 | -0.045 | -0.079, -0.011 | 0.009 |
| On TDF versus not on TDF | -0.023 | -0.065, 0.019 | 0.272 | - | - | - |
| Cumulative exposure to ART (years)  Cumulative exposure to PI  Cumulative exposure to TDF | -0.006  -0.009  -0.005 | -0.011, -0.001  -0.016, -0.002  -0.014, 0.004 | 0.017  0.007  0.292 | -0.005  -0.004  - | -0.010, 0.0002  -0.010, 0.004  - | 0.06  0.33  - |
| Number of ART regimens | -0.018 | -0.028, -0.009 | 0.0002 | -0.005 | -0.018, 0.0008 | 0.07 |

**Supplementary Table 2: Unadjusted and Adjusted analysis within the HIV positive subgroup exploring associations between HIV-specific factors and lower TBS**

TBS – Trabecular bone score, ART – Antiretroviral therapy, C.I – Confidence Interval, IVDU – Intravenous drug use, PI – Protease inhibitor, TDF – Tenofovir disproxil fumarate

Table legend: *All variables adjusted for age, gender, Ethnicity, body mass index and current smoking status. Variables which remained significant following these adjustments remained in subsequent models until association was lost and the final model which best predicted lower TBS was reached, i.e All variables after nadir CD4T-cell count had this variable in the model, IVDU remained in the models until the inclusion of PI exposure and was excluded from subsequent models as the association was lost, PI exposure was included in all models subsequent to it’s inclusion.

^ IVDU versus non IVDU adjusted for age, gender, Ethnicity, body mass index and current smoking status, nadir CD4 T-cell count and On Protease Inhibitor
