# Supplementary material for: Assessment of trabecular bone score, an index of bone microarchitecture, in HIV positive and HIV negative persons within the HIV UPBEAT cohort
Source: PLoS One. 2019 Mar 21;14(3):e0213440. doi: 10.1371/journal.pone.0213440 (PMC6428393; doi:10.1371/journal.pone.0213440)
Supplement: S1 File — Trabecular Bone Score (Median [IQR]); Between-group comparisons (Table A). Unadjusted HIV Effect on Trabecular Bone Score (Table B). Multivariable Models of Independent predictors of TBS (Models i to viii) (Table C). Multivariable model: Predictors of lower TBS using duration of smoking in years as a surrogate for tobacco consumption (Table D). (DOCX) [file pone.0213440.s003.docx]

**Supplementary Table A in S1 File : Trabecular Bone Score (Median [IQR]); Between-group comparisons**

| Group | n | TBS Median [IQR] | *P** |
| --- | --- | --- | --- |
| HIV positive  HIV negative | 201  262 | 1.349 [1.263, 1.436]  1.380 [1.301, 1.453] | 0.009 |
| Male  Female | 233  230 | 1.346 [1.274, 1.435]  1.380 [1.310, 1.454] | 0.045 |
| African  Non- African | 114  319 | 1.386 [1.301, 1.457]  1.363 [1.279, 1.438] | 0.038 |
| Education:  *3^rd^ level Education*  *<3^rd^ level Education* | 169  267 | 1.380 [1.298, 1.454]  1.359 [1.270, 1.434] | 0.024 |
| Current Smoker  Non current smoker  Current smoker  Never smoker  Current smoker  Ex smoker  Never smoker  Ex smoker | 116  347  116  267  116  80  267  80 | 1.335 [1.230, 1.409]  1.380 [1.304, 1.456]  1.335 [1.230, 1.409]  1.382 [1.310, 1.460]  1.335 [1.230, 1.409]  1.361 [1.290, 1.421]  1.382 [1.310, 1.460]  1.361 [1.290, 1.421] | <0.0001  <0.0001  0.04  0.08 |
| Excess Alcohol Intake  No Excess Alcohol Intake | 49  414 | 1.329 [1.262, 1.395]  1.372 [1.289, 1.451] | 0.05 |
| Low LS BMD  Normal LS BMD | 49  414 | 1.301 [1.142, 1.387]  1.379 [1.298, 1.453] | <0.0001 |
| Prior fracture  No prior fracture | 124  339 | 1.338 [1.257, 1.421]  1.380 [1.303, 1.454] | 0.002 |

*P values calculated using Mann-Whitney Wilcoxon tests

| (i). Variable | Effect on TBS | 95% C.I | *P* |
| --- | --- | --- | --- |
| HIV status | -0.037 | -0.060,-0.013 | 0.002 |

**Supplementary Table C: Multivariable Models of Independent predictors of TBS (Models i to viii)**

**Supplementary Table B in S1 File: Unadjusted HIV Effect on Trabecular Bone Score**

| (ii). Variable | Effect on TBS | 95% C.I | *P* |
| --- | --- | --- | --- |
| HIV status | -0.037 | -0.060, -0.013 | 0.002 |
| LS BMD (per 0.01g/cm^2^ increase) | 0.003 | 0.184, 0.321 | <0.0001 |

| (iii). Variable | Effect on TBS | 95% C.I | *P* |
| --- | --- | --- | --- |
| HIV status | -0.029 | -0.053, -0.006 | 0.01 |
| LS BMD (per 0.01g/cm^2^ increase) | 0.002 | 0.002, 0.003 | <0.0001 |
| Age (per 5 year increase) | -0.014 | -0.020, -0.009 | <0.0001 |
| Gender | -0.0005 | -0.024, 0.023 | 0.96 |
| Ethnicity (African versus non African) | 0.0004 | -0.027, 0.028 | 0.97 |
| BMI (per 10kg/m^2^ increase) | 0.006 | -0.017, 0.030 | 0.59 |

| (iv). Variable | Effect on TBS | 95% C.I | P |
| --- | --- | --- | --- |
| HIV status | -0.018 | -0.042, 0.006 | 0.132 |
| BMD lumbar spine (per 0.01 gm/cm2 increase) | 0.002 | 0.002, 0.003 | <.0001 |
| Age (per 5 year older) | -0.015 | -0.021, -0.009 | <.0001 |
| Gender | 0.002 | -0.022, 0.025 | 0.876 |
| Ethnicity (African versus non African) | -0.013 | -0.041, 0.015 | 0.371 |
| BMI (per 10 kg/m2 increase) | 0.0006 | -0.022, 0.024 | 0.958 |
| Current vs non-current smoker | -0.054 | -0.081, -0.027 | <.0001 |

| (v). Variable | Effect on TBS | 95% C.I | *P* |
| --- | --- | --- | --- |
| HIV status | 0.001 | -0.024, 0.027 | 0.96 |
| BMD LS (per 0.01g/cm^2^ increase) | 0.200 | 0.130, 0.270 | <0.0001 |
| Age (per 5 year older) | -0.014 | -0.020, -0.008 | <0.0001 |
| Gender | -0.0004 | -0.025, 0.024 | 0.97 |
| Ethnicity (African versus non African) | -0.014 | -0.042, 0.014 | 0.32 |
| BMI (per 10kg/m^2^ increase) | 0.007 | -0.015, 0.031 | 0.50 |
| Current versus non current smoker | -0.041 | -0.067, -0.013 | 0.004 |
| Albumin (per 5g/L) | 0.019 | 0.002, 0.036 | 0.03 |
| Alk Phosphatase (per 5 IU/L) | -0.004 | -0.006, -0.002 | 0.0002 |

| (vi). Variable | Effect on TBS | 95% C.I | *P* |
| --- | --- | --- | --- |
| HIV status | -0.006 | -0.031, 0.020 | 0.66 |
| BMD LS (per 0.01g/cm^2^ increase) | 0.214 | 0.141, 0.288 | <0.0001 |
| Age (per 5 year older) | -0.014 | -0.020, -0.008 | <0.0001 |
| Gender | -0.006 | -0.030, 0.019 | 0.66 |
| BMI (per 10kg/m^2^ increase) | 0.004 | -0.020, 0.027 | 0.75 |
| Ethnicity (African versus non African) | -0.014 | -0.043, 0.015 | 0.33 |
| Current versus non current smoker | -0.040 | -0.068, -0.013 | 0.005 |
| Albumin (per 5g/L) | 0.027 | 0.010, 0.044 | 0.002 |
| OC | -0.004 | -0.010, 0.002 | 0.18 |

| (vii). Variable | Effect on TBS | 95% C.I | *P* |
| --- | --- | --- | --- |
| HIV status | -0.006 | -0.031, 0.019 | 0.65 |
| BMD LS (per 0.01g/cm^2^ increase) | 0.216 | 0.144, 0.289 | <0.0001 |
| Age (per 5 year older) | 0.015 | -0.021,-0.009 | <0.0001 |
| Gender | -0.003 | -0.0283, 0.022 | 0.79 |
| Ethnicity (African versus non African) | -0.010 | -0.038, 0.018 | 0.48 |
| Current versus non current smoker | -0.040 | -0.068, -0.012 | 0.005 |
| BMI (per 10kg/m^2^ increase) | 0.003 | -0.019, 0.027 | 0.75 |
| Albumin (per 5g/L) | 0.026 | 0.009, 0.043 | 0.002 |
| P1NP | -0.004 | -0.008, 0.0002 | 0.06 |

| (viii). Variable | Effect on TBS | 95% C.I | *P* |
| --- | --- | --- | --- |
| HIV status | -0.005 | -0.031, 0.019 | 0.66 |
| BMD LS (per 0.01g/cm^2^ increase) | 0.211 | 0.137, 0.285 | <0.0001 |
| Age (per 5 year older) | -0.014 | -0.020, -0.008 | <0.0001 |
| Gender | -0.004 | -0.029, 0.021 | 0.76 |
| Ethnicity (African versus non African) | -0.013 | -0.043, 0.015 | 0.34 |
| Current versus non current smoker | 0.042 | -0.071, -0.015 | 0.002 |
| BMI (per 10kg/m^2^ increase) | 0.003 | -0.021, 0.027 | 0.80 |
| Albumin (per 5g/L) | 0.026 | 0.009, 0.043 | 0.002 |
| CTx | -0.004 | 0.010, 0.001 | 0.133 |

**Supplementary Table D in S1 File: Multivariable model: Predictors of lower TBS using duration of smoking in years as a surrogate for tobacco consumption.**

| Variable | Effect on TBS | 95% C.I | P |
| --- | --- | --- | --- |
| HIV status | -0.015 | -0.071, 0.005 | 0.258 |
| BMD lumbar spine (per 0.01 gm/cm2 increase) | 0.003 | 0.002, 0.005 | <.0001 |
| Age (per 5 year older) | -0.018 | -0.025, -0.015 | <.0001 |
| Gender | 0.002 | -0.028, 0.104 | 0.853 |
| Ethnicity (African versus non African) | -0.012 | -0.034, 0.029 | 0.876 |
| BMI (per 10 kg/m2 increase) | 0.006 | -0.010, 0.040 | 0.452 |
| Duration of smoking (per 5 year increase) | -0.027 | -0.035, 0.005 | 0.167 |
